# 

**Published:** 1870-11

**Authors:** 


### Books and Pamphlets Received.

The Pathology and Treatment of Venereal Diseases. By Freeman J. Bumstead, M. D.. Professor of Venereal Diseases at the College of Physicians and Surgeons, New York, etc. Third edition, revised, enlarged and illustrated. Philadelphia: H. C. Lea. For sale by T. Butler & Son.

Transactions of the American Medical Association. Volume XXI.

Practical Anatomy: A Manual of Dissections. By Christopher Heath, F. R. C. S. First American from second English edition. Edited by William W. Keen, M. D., Lecturer on Pathological Anatomy in Jefferson Medical College, etc. Philadelphia: H. C. Lea. For sale by T. Butler & Son.

The Medical Adviser. By Rezin Thompson, M. D. Cincinnati: National Publishing Co.

Transactions of the Medical Society of the State of West Virginia, at its Third Annual Meeting, held in Parkersburg, June, 1870.

Syphilis of the Nervous System: A Clinical Study in regard to Diagnosis and Treatment. By E. L. Keyes, M. D., Physican to the Bureau of Ont-door Relief Bellevue Hospital.

Observations on the Effects of Galvanization of the Sympathetic. By A. D. Rockwell, A. M., M. D., and Geo. M. Beard, A. M., M. M., New York.

Note on the Value of Wheat Phosphates in Therapeusis. By J. S. Hawley, A. M., M. D., Greenpoint, N. Y.

Constitution and By Laws of the Medical Library and Journal Association, of New York.

Illustrated Catalogue of the Publications of Henry C. Lea, Philadelphia.

Catalogue of Medical Books for sale by Wm. Wood & Co., New York.

Catalogue of the Publications of Lindsay & Blakiston, Philadelphia.

Illustrated Annual of Phrenology and Physiognomy. S. R. Wells, New York.

Prescription and Clinic Record. Wm. Wood & Co., New York.

